# Presentation of life-threatening invasive nontyphoidal *Salmonella* disease in Malawian children: A prospective observational study

**DOI:** 10.1371/journal.pntd.0006027

**Published:** 2017-12-07

**Authors:** Calman A. MacLennan, Chisomo L. Msefula, Esther N. Gondwe, James J. Gilchrist, Paul Pensulo, Wilson L. Mandala, Grace Mwimaniwa, Meraby Banda, Julia Kenny, Lorna K. Wilson, Amos Phiri, Jenny M. MacLennan, Elizabeth M. Molyneux, Malcolm E. Molyneux, Stephen M. Graham

**Affiliations:** 1 Jenner Institute, Nuffield Department of Medicine, University of Oxford, Oxford, United Kingdom; 2 School of Immunity and Infection, College of Medicine and Dental Sciences, University of Birmingham, Birmingham, United Kingdom; 3 Malawi-Liverpool-Wellcome Trust Clinical Research Programme, College of Medicine, University of Malawi, Malawi; 4 Department of Microbiology, College of Medicine, University of Malawi, Malawi; 5 Liverpool School of Tropical Medicine, Pembroke Place, Liverpool, United Kingdom; 6 Department of Biochemistry, College of Medicine, University of Malawi, Malawi; 7 Department of Paediatrics, University of Oxford, United Kingdom; 8 Wellcome Trust Centre for Human Genetics, University of Oxford, United Kingdom; 9 Department of Basic Medical Sciences, College of Medicine, University of Malawi, Blantyre, Malawi; 10 Academy of Medical Sciences, Malawi University of Science and Technology, Thyolo, Malawi; 11 Department of Paediatrics, College of Medicine, University of Malawi, Malawi; 12 Infectious Diseases and Microbiology Unit, Institute of Child Health, University College London, London, United Kingdom; 13 Department of Zoology, University of Oxford, Oxford, United Kingdom; 14 Department of Medicine, College of Medicine, University of Malawi, Malawi; 15 Centre for International Child Health, University of Melbourne and Murdoch Children’s Research Institute, Royal Children’s Hospital, Melbourne, Australia; Liverpool School of Tropical Medicine, UNITED KINGDOM

## Abstract

Nontyphoidal *Salmonellae* commonly cause invasive disease in African children that is often fatal. The clinical diagnosis of these infections is hampered by the absence of a clear clinical syndrome. Drug resistance means that empirical antibiotic therapy is often ineffective and currently no vaccine is available. The study objective was to identify risk factors for mortality among children presenting to hospital with invasive *Salmonella* disease in Africa. We conducted a prospective study enrolling consecutive children with microbiologically-confirmed invasive *Salmonella* disease admitted to Queen Elizabeth Central Hospital, Blantyre, in 2006. Data on clinical presentation, co-morbidities and outcome were used to identify children at risk of inpatient mortality through logistic-regression modeling. Over one calendar year, 263 consecutive children presented with invasive *Salmonella* disease. Median age was 16 months (range 0–15 years) and 52/256 children (20%; 95%CI 15–25%) died. Nontyphoidal serovars caused 248/263 (94%) of cases. 211/259 (81%) of isolates were multi-drug resistant. 251/263 children presented with bacteremia, 6 with meningitis and 6 with both. Respiratory symptoms were present in 184/240 (77%; 95%CI 71–82%), 123/240 (51%; 95%CI 45–58%) had gastrointestinal symptoms and 101/240 (42%; 95%CI 36–49%) had an overlapping clinical syndrome. Presentation at <7 months (OR 10.0; 95%CI 2.8–35.1), dyspnea (OR 4.2; 95%CI 1.5–12.0) and HIV infection (OR 3.3; 95%CI 1.1–10.2) were independent risk factors for inpatient mortality. Invasive *Salmonella* disease in Malawi is characterized by high mortality and prevalence of multi-drug resistant isolates, along with non-specific presentation. Young infants, children with dyspnea and HIV-infected children bear a disproportionate burden of the *Salmonella*-associated mortality in Malawi. Strategies to improve prevention, diagnosis and management of invasive *Salmonella* disease should be targeted at these children.

## Author summary

Nontyphoidal *Salmonellae* are a major, yet often neglected, cause of fatal invasive disease among young African children, responsible for over 100,000 deaths a year. There is currently a lack of prospective studies to understand the clinical presentation and course of this disease. Diagnosis can be confusing, antibiotic resistance is an increasing problem and no vaccine is available. Studying 263 consecutive cases of invasive *Salmonella* disease over a one year period, we observed disparate clinical presentations, precluding the possibility of making a reliable clinical diagnosis in cases of this disease. We confirmed high mortality rates, finding that young age, dyspnea and HIV infection are independent risk factors for death. These findings support both the need for improved measures for prevention, diagnosis and management of invasive nontyphoidal *Salmonella* disease and indicate which children with this disease are at most need of intensive clinical care.

## Introduction

Invasive bacterial disease is a major cause of mortality among African children [1–4]. Prospective studies from rural-based district hospitals in Kenya and Mozambique have found community-acquired bacteremia to be responsible for 26% [1] and 21% [3] of pediatric inpatient deaths. Pneumococcus and nontyphoidal *Salmonellae* (NTS) are the most common causes of bacteremia among African children [1–5]. While the burden of disease due to pneumococcus should fall with implementation of pneumococcal conjugate vaccines, no vaccine is available or in clinical trials against invasive nontyphoidal *Salmonella* (iNTS) disease [6, 7].

iNTS disease among African children is caused primarily by *Salmonella enterica* serovars Typhimurium and Enteritidis [8–12], and has an associated case fatality of around 20–25% for bacteremia [8, 9, 11, 13, 14] and 52% for meningitis [15]. These findings contrast with *Salmonella* disease in industrialized nations which typically presents as self-limiting enterocolitis, rarely requiring hospitalization [16]. Despite reports of declining iNTS disease with falling prevalence of malaria from specific African sites [17–19], recently published data from multiple countries across sub-Saharan Africa indicate that NTS continues to be a major cause of childhood bacteremia in west [20,21], central [22], east [23,24] and southern Africa [25,26]. The Phase 3 RTS,S/AS01 malaria vaccine trial conducted in eleven sites across sub-Saharan Africa, agnostic to levels of *Salmonella* bacteremia, found an incidence of approximately 500 cases of *Salmonella* sepsis/100,000 children/year among children under two years [27]. Recent data from the Typhoid Surveillance in Africa Program across a further seven sub-Saharan African sites confirm that iNTS disease continues to be a major public health problem in the region [28].

The clinical presentation of iNTS disease is often a non-specific febrile illness [7–10, 14]. Further diagnostic challenges result from the association of iNTS disease with other co-morbidities [7–10, 14]. Among African children, iNTS disease often occurs with malaria [29–31], HIV [26, 31], malnutrition [32] and anemia [33]. In adults, there is a strong association with HIV infection [26, 34]. Increasing multi-drug resistance among iNTS isolates in Africa adds to the high attrition from iNTS disease [8, 10–12]. For Malawian isolates, 90% have been reported as resistant to ampicillin, chloramphenicol and cotrimoxazole [35].

We set out to provide data to guide improved management of children with iNTS disease. Consecutive microbiologically-confirmed cases admitted to a large government hospital in Malawi were studied for the relationship between clinical presentation and outcome.

## Methods

### Study site

Queen Elizabeth Central Hospital (QECH) is the largest hospital in Malawi. It is situated in Blantyre, one of the two main cities in Malawi, and serves a combined urban and peri-urban rural population of around 1 million. The hospital has approximately 1000 beds and admits around 10,000 adults and 30,000 children (<16 years) annually. Since 1996, the Malawi-Liverpool-Wellcome Trust Clinical Research Programme has performed blood and cerebrospinal fluid (CSF) cultures from patients admitted with suspected sepsis and meningitis.

### Study patients and clinical methods

Eligible participants were children age ≤ 14 years admitted to the Pediatric Department of QECH with isolation of *Salmonella* from blood and/or CSF between January 1st and December 31st 2006. Subjects not meeting all three eligibility criteria were excluded. In order to reduce bias due to early death following admission, children were approached for recruitment on the day that Gram-negative bacteria were first detected in the blood or CSF, either on initial Gram stain or following positive culture.

First-line antimicrobial therapy for suspected sepsis in children at the time of the study was intramuscular chloramphenicol and gentamicin, or penicillin and gentamicin. The great majority of Gram-negative bacteraemias detected were due to NTS, and these bacteria were almost all resistant to chloramphenicol and penicillin. Therefore, children were started on oral ciprofloxacin and/or intravenous ceftriaxone as soon as Gram-negative bacteria were first detected. Once the organism and antibiotic sensitivity profile were known, treatment would be amended as necessary.

First line antimicrobial therapy for pediatric meningitis was intravenous ceftriaxone. Demographic and clinical data collected on admission were recorded on standard forms, along with weight and height or length. Clinical progress was recorded daily through to discharge from hospital or inpatient death.

Respiratory distress was defined as the presence of a tracheal tug, intercostal or subcostal recession, head-bobbing or nasal flaring. Tachypnea and tachycardia on admission were defined as rates greater than the 90th centile for age [36]. Children with an admission axillary temperature >37.5°C were classified as febrile. Children were considered to have gastroenteritis if diarrhea (≥3 loose stools per day) or vomiting were present, and respiratory disease if there was shortness of breath or respiratory distress. Fever without focus was defined as fever in the absence of localizing clinical signs.

Severe malnutrition in children 6–60 months was defined as a weight-for-height greater than 3 z-scores below the WHO median, or bilateral pedal edema (kwashiorkor). In children over 60 months, severe malnutrition was defined as a body mass index (BMI)-for-age greater than 3 z-scores below the WHO median. Severe anemia was defined as a packed cell volume (PCV) <15% or a hemoglobin concentration <5.0 g/dl. Hypoglycemia was defined as a glucose concentration on admission <3.0 mmol/L.

### Sampling and laboratory methods

Blood and CSF cultures were performed using a standard aerobic bottle (BacT/Alert, bioMérieux, France) using 1–2 ml of blood and 0.5–1 ml of CSF. Following detection of Gram-negative bacteria, *Salmonellae* were confirmed by biochemical testing with API 20E kits (bioMérieux) and serovar was determined using agglutinating antisera (Prolab Diagnostics). Antimicrobial susceptibility testing was performed by disc diffusion using ampicillin, chloramphenicol, cotrimoxazole, gentamicin, ceftriaxone and ciprofloxacin discs according to British Society of Antimicrobial Chemotherapy methods and breakpoints. The laboratory participates in the UK National External Quality Assurance Scheme. All children admitted to QECH have a malaria parasite slide and PCV performed. Full blood counts were determined using a HMX (Becton Coulter). Tests for HIV infection were with Determine (Abbot Laboratories) and UniGold (Trinity Biotech) rapid tests, following national Voluntary Counseling and Testing guidelines. For discordant results and children under 18 months with positive results, the diagnosis was confirmed by polymerase chain reaction for proviral DNA.

### Rainfall data

Monthly rainfall data through 2006 were collected from the meteorological station at Chileka Airport, Blantyre.

### Statistical analysis

Data were entered into a Microsoft Access database and analysis was performed using R (http://www.R-project.org/). Variation with age and season, of clinical indices, co-morbidity and outcome, were calculated as Mantel-Haenszel odds ratios. To facilitate this, age was converted into four ordinal categories (0–6 months, 7–12 months, 1–2 years, >2 years). Presentation season was grouped as January-March, April-June, July-September and October-December and ranked by mean rainfall. Odds ratios of case fatality given the presence of clinical indices or co-morbidity adjusted for age and season of presentation were calculated by logistic regression. Finally, a model of case fatality was constructed and tested by multivariate logistic regression. Covariates were chosen for the first fit of the model if they predicted mortality with a p value <0.1 along with age and sex. To minimize the number of covariate patterns, age was collapsed into a binary variable (children 0–6 months and children 7 months and over) for the purposes of the model. Covariates with the largest remaining p value (but retaining age and sex) were sequentially removed from the model and the model re-evaluated for goodness-of-fit and distribution of residuals at each step. The model was also evaluated for two-way interactions between significantly-associated terms. Missing data were addressed by providing denominator information where applicable.

### Ethics statement

After ensuring that each child was on appropriate antimicrobial therapy, the study was explained to the parents or guardians and informed written consent obtained. Ethical approval for the study was granted by the College of Medicine Research and Ethics Committee, College of Medicine, University of Malawi.

## Results

**Fig 1:**
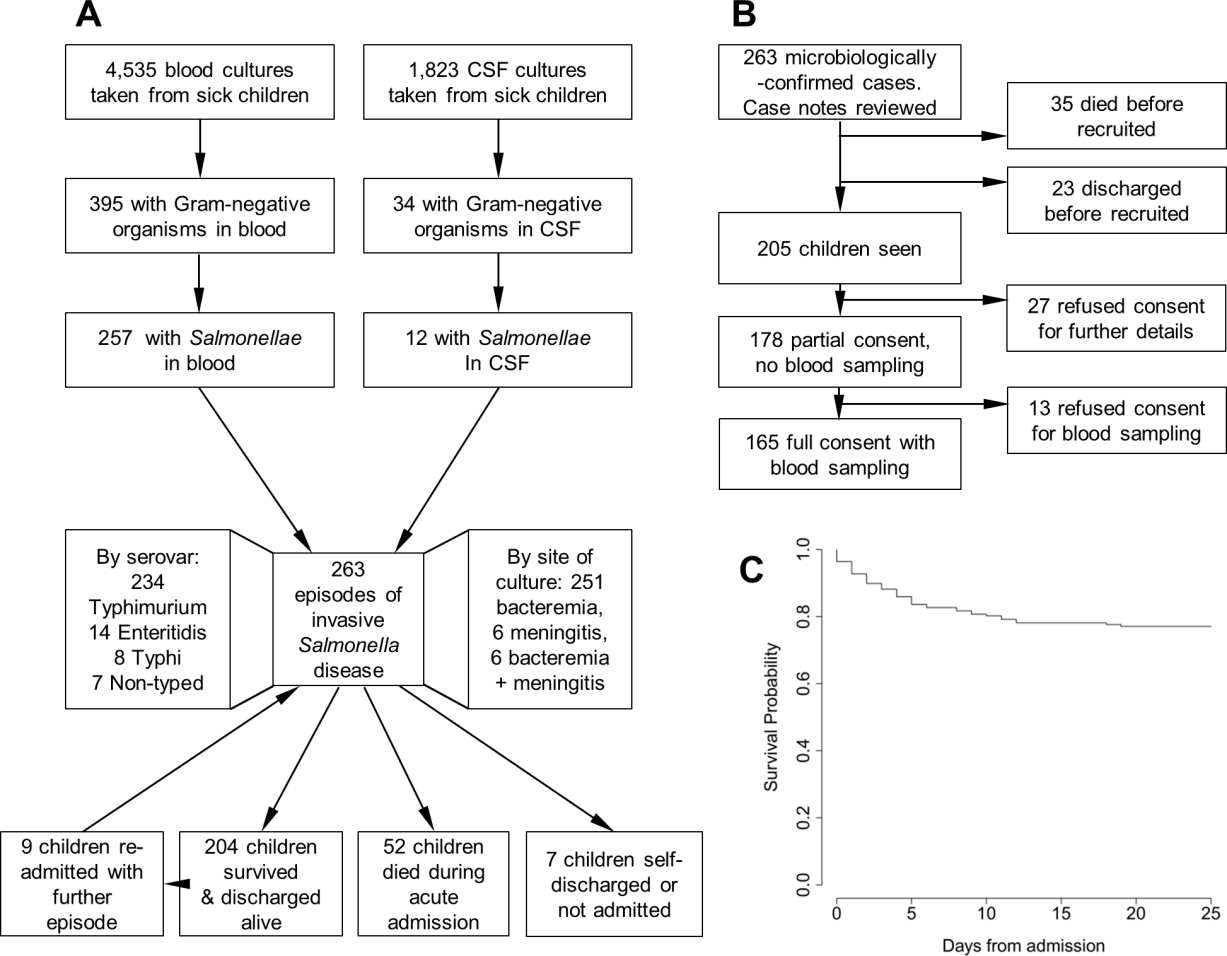
Malawian children admitted to hospital with invasive *Salmonella* disease, recruitment and outcomes. (A) Blood and CSF cultures taken from children admitted to Queen Elizabeth Central Hospital, Blantyre, Malawi in 2006; those yielding *Salmonella* isolates; and outcomes of children with invasive *Salmonella* disease. (B) Recruitment pathway for children following detection of Gram-negative bacteria in blood and/or CSF culture. (C) Kaplan-Meier estimate of survival (in days) of children following admission with invasive *Salmonella* disease.

In total, 4535 blood cultures and 1823 CSF cultures were performed on children admitted to QECH over one year in 2006. Gram-negative organisms were detected in 395/4535 (9%) blood cultures and 34/1823 (2%) CSF cultures. Of these, 257/395 (65%) and 12/34 (35%), respectively, were identified as *Salmonellae* (Fig 1A). *Salmonellae* were isolated from both blood and CSF of six children, giving a total of 263 episodes of invasive *Salmonella* disease. The majority of *Salmonellae* 234/263 (89%) were serovar Typhimurium, while 14/263 (5%) were serovar Enteritidis and only 8/263 (3%) Typhi. 7/263 (3%) could not be typed with locally available antisera. Nine children had either a single (n = 5) or double (n = 4) recurrence of NTS bacteremia. NTS bacteraemia occurred in two sets of twins.

**Fig 2:**
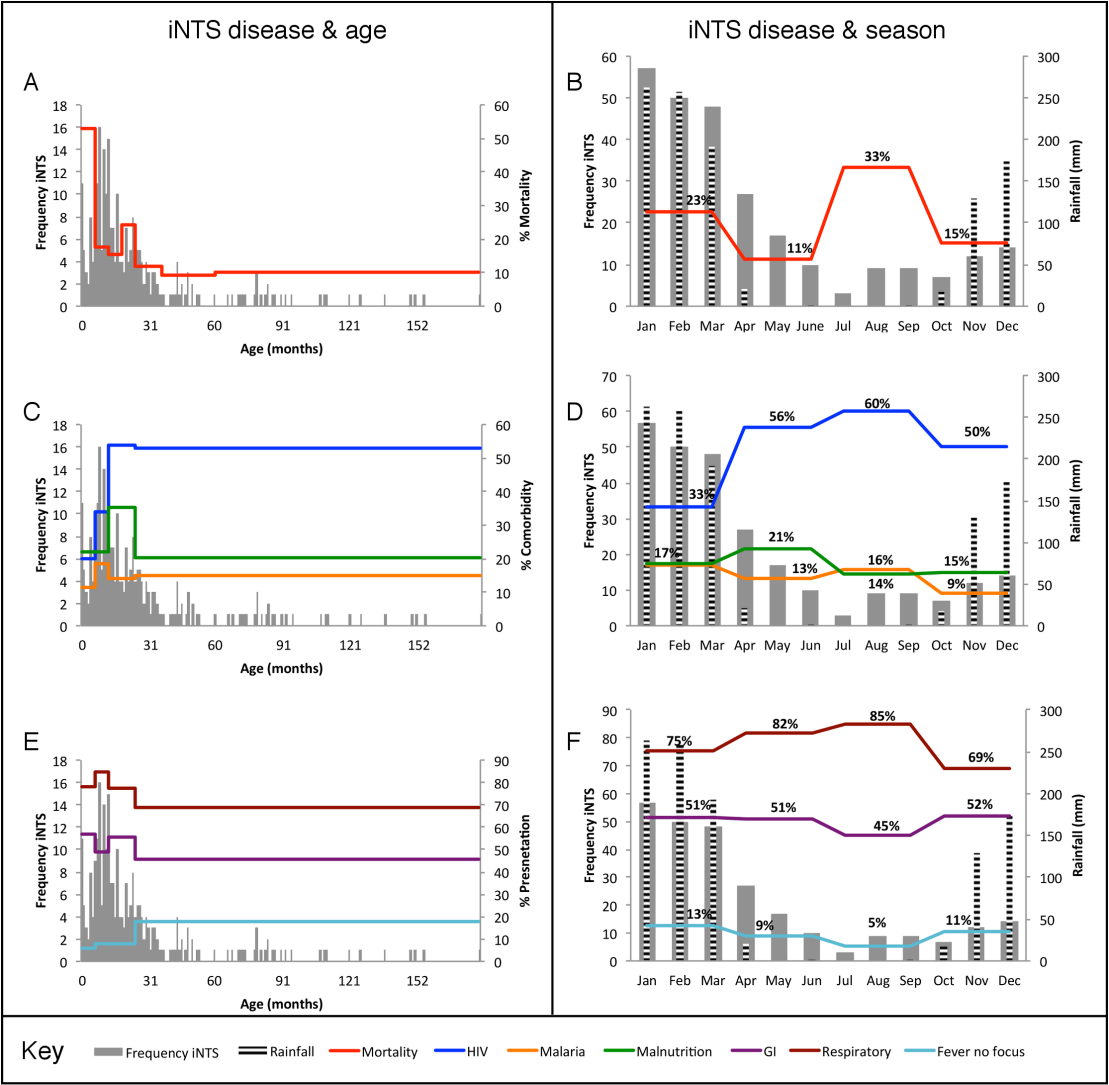
Variability in outcome, co-morbidity and clinical presentation of invasive *Salmonella* disease in Malawian children with age and season of presentation. Graphs indicate variation in mortality (A and B), co-morbidity (C and D) and clinical presentation (E and F) with age and month of the year. Each plot is overlaid on a histogram of the frequency of iNTS disease presentation with age (A, C and E) or bar-charts indicating monthly frequency of iNTS disease and monthly rainfall (B, D and F).

Of the 263 children with microbiologically-confirmed invasive *Salmonella* disease, 35 (13%) had died and 23 (9%) been discharged prior to recruitment, leaving 205 (78%) whose parents and guardians were approached for consent. Consent was obtained for 178 (87%) out of the 205 children seen (Fig 1B). Median age of children was 16 months (range 0 months to 15 years) with 213/263 (81%) under 3 years (Table 1 and Fig 2A). The incidence of disease was associated significantly with rainfall (linear regression coefficient 4.70, 95% CI 2.48–6.92, p = 0.001), with 152/263 (58%) presenting between January and March during the rainy season (which last from November to April), and 30/263 from June to September in the dry season (11%) (Fig 2B). 7/263 (3%) children self-discharged or were not admitted.

**Table 1:** Characteristics of children presenting with invasive *Salmonella* disease.

Age (months), median (range)	16 (0–180)	
	Number	Percentage (95% CI)
Male	144/263	54.8 (48.7–60.8)
Inpatient mortality	52/256	20.3 (15.4–25.3)
HIV	70/162	43.2 (35.5–50.9)
Severe malnutrition	45/180	25.0 (18.6–31.4)
Malaria	38/249	15.3 (10.8–19.8)
MDR isolate	211/259	81.5 (76.7–86.2)

Abbreviations: CI, confidence interval; MDR, multidrug resistant.

### Clinical presentation

Clinical features and comorbidities of children with invasive *Salmonella* disease are shown in Table 2 and Fig 2. Most children presented with a history of fever and were febrile on admission. Cough was significantly more common as a presenting symptom than either diarrhea (p<0.001) or vomiting (p<0.001), with signs of respiratory distress in 72/166 (43%) of children at admission. Older children (≥7 months) tended to present more often with fever in isolation than younger children (<7 months; OR = 1.26, 95% CI 1.02–2.20). Neither the proportions of respiratory nor gastrointestinal presentations varied with age (Fig 2E), and clinical presentation did not vary significantly with season of presentation (Fig 2F).

**Table 2:** Clinical characteristics of Malawian children with invasive *Salmonella* disease and associated mortality.

			Risk of death^a^	
		Number (%)	Odds Ratio (95% CI)	p value
**Symptoms**				
	Fever	229/243 (94.2)	0.21 (0.06–0.69)	**0.010**
	Convulsions	22/234 (9.4)	0.86 (0.23–3.13)	0.813
	Vomiting	91/241 (37.8)	1.38 (0.70–2.72)	0.347
	Diarrhea	75/241 (31.1)	1.70 (0.86–3.39)	0.128
	Cough	167/234 (71.4)	1.83 (0.78–4.33)	0.163
	Dyspnea	96/228 (42.1)	3.97 (1.90–8.31)	**<0.001**
**Signs**				
	Febrile	184/227 (81.1)	0.62 (0.27–1.41)	0.257
	Tachycardia^b^	46/67 (68.7)	2.30 (0.54–9.78)	0.261
	Tachypnea^b^	50/76 (65.8)	1.95 (0.54–7.01)	0.305
	BCS<5	25/231 (10.8)	2.23 (0.86–5.80)	0.099
	Dehydration	24/222 (10.8)	1.22 (0.45–3.33)	0.697
	Candida	42/234 (17.9)	0.86 (0.35–2.11)	0.741
	Respiratory distress	72/166 (43.4)	3.42 (1.48–7.89)	**0.004**
	Hepatomegaly	52/232 (22.4)	1.21 (0.53–2.73)	0.649
	Splenomegaly	62/231 (26.8)	0.51 (0.21–1.23)	0.137
**Investigations**				
	Severe anemia	47/244 (19.3)	0.94 (0.40–2.22)	0.884
	Hypoglycemia	9/49 (18.4)	12.20 (2.04–72.78)	**0.006**
**Co-morbidity**				
	Malaria	38/249 (15.3)	0.75 (0.29–1.97)	0.565
	HIV	70/162 (43.2)	3.56 (1.25–10.08)	**0.017**
	Severe Malnutrition	45/180 (25.0)	1.93 (0.74–5.03)	0.176
**Pathogen**				
	MDR isolate	211/259 (81.5)	1.07 (0.45–2.61)	0.888

^a^Adjusted for age and season.

^b^Estimates of children with tachypnea and tachycardia are based on the small numbers of children for which respiratory rates and heart rates were documented on presentation (n = 76 & 67).

CI denotes confidence interval.

Abbreviations: BCS, Blantyre Coma Score; MDR, multidrug resistant.

45/180 (25%) of patients were severely malnourished. Median PCV on admission was 25%, and 47/244 (19%) of children were severely anemic. 38/249 (15%) had concurrent *Plasmodium falciparum* parasitemia. Of children tested, 70/162 (43%) were HIV-infected. Significantly more older children (≥7 months) were HIV-infected than younger children (<7 months, OR = 1.55; 95% CI 1.15–2.09). Malaria and malnutrition did not vary significantly with age (Fig 2C). Children diagnosed with iNTS disease during the months with high rainfall were less likely to be HIV-infected than those admitted in the dry season (OR = 0.67; 95% CI 0.49–0.89). The rates of malaria and malnutrition did not vary significantly with season (Fig 2D).

### Antimicrobial resistance and usage

Most strains were resistant to ampicillin, cotrimoxazole and chloramphenicol (multi-drug resistant; 211/259, 81.5%). None were resistant to ciprofloxacin or ceftriaxone. Only 15/244 (6%) of children received first-line antimicrobials on admission to which their *Salmonella* isolate was susceptible in vitro. Conversion to either ceftriaxone or ciprofloxacin occurred with a median delay of 3 days (range 1–12 days).

### Mortality

Inpatient mortality was 52/256 (20%) (Fig 1C), highest among infants of 0 to 6 months inclusive (53%) (Fig 2A), and declined with increasing age (OR = 0.56, 95%CI 0.42–0.75; age in this model was divided into four ordinal categories, children 0–6 months, 7–12 years, 13–24 months, and >24 months). Mortality did not vary significantly with season (Fig 2B). Death occurred on the calendar day of admission in 8/52 (15%) of children who died, with a median time from admission to death of 3 days (range 0–18 days). Adjusting for age and season, significant risk factors for mortality were: a history of dyspnea, absence of fever, presence of respiratory distress or hypoglycemia at presentation, and HIV infection (Table 2). Cotrimoxazole preventive therapy (CPT) was not associated with mortality in HIV-infected children (OR = 2.17, 95%CI = 0.54–10.88; p = 0.30). 147/204 (72%) of children surviving their acute admission were followed up four to six weeks post-discharge from hospital (median time to follow up: 43 days), with no reported deaths in the intervening time. Only one out of the nine children with recurrent NTS bacteremia died.

**Fig 3:**
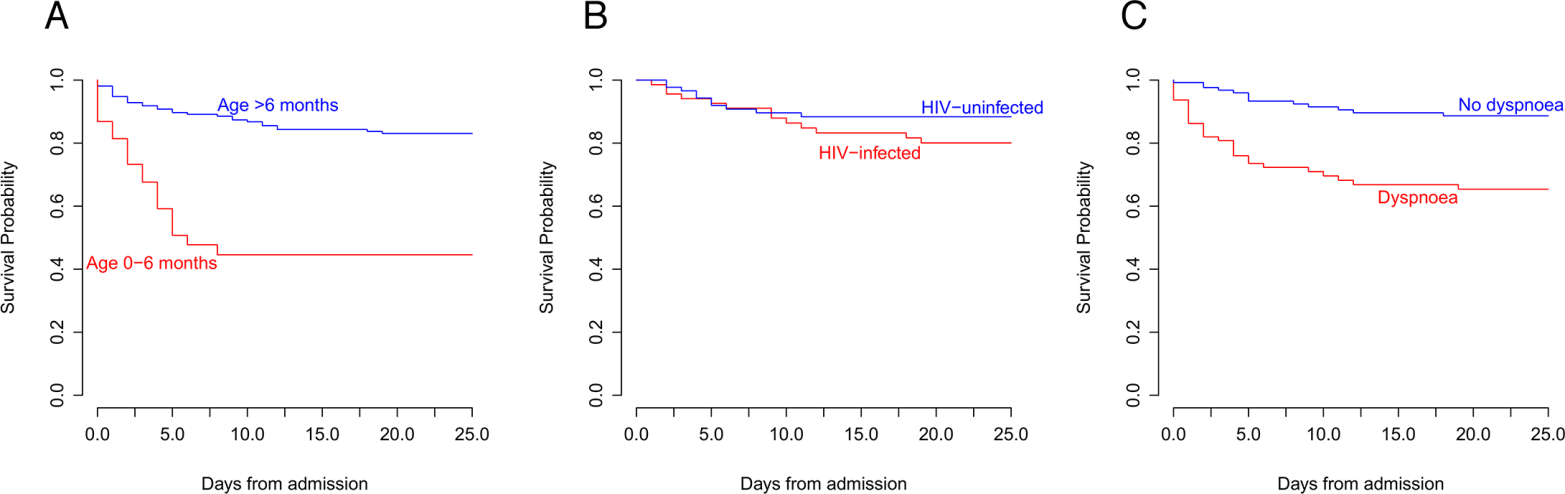
Survival of children with invasive *Salmonella* disease. Kaplan-Meier estimates of survival (in days) following admission with invasive *Salmonella* disease in children aged 0–6 months and >6 months (A), children with and without HIV co-infection (B), and children with and without a history of dyspnea at presentation (C).

A logistic regression model predicting mortality including age, sex, HIV status and a history of dyspnea was shown to be statistically significant (likelihood ratio test χ^2^ = 21.69, p = 0.0002) with a pseudo-R^2^ of 0.179. The Hosmer-Lemeshow goodness-of-fit test for the model provides no evidence to reject the model (p = 0.73) and the distribution and influence of the residuals (S1 Fig) demonstrate that the model is consistent with the data. In this model, age less than 7 months (OR = 10.0; 95% CI 2.8–35.1), HIV infection (OR = 3.3, 95% CI 1.1–10.2) and a history of dyspnea (OR = 4.2; 95% CI 1.5–12.0) were significant independent risk factors for death. Inpatient survival in children with and without each of these risk factors for mortality is presented in Fig 3.

## Discussion

*Salmonellae* are a common cause of invasive disease in African children that is often fatal. The clinical features and high mortality in the current study are unchanged from a retrospective study from the same hospital a decade earlier [37]. Almost all patients had nontyphoidal serovars, most commonly Typhimurium, and the majority of isolates were MDR. In some settings, particularly urban centers, typhoid fever is more common than iNTS disease [12,24].

**Fig 4:**
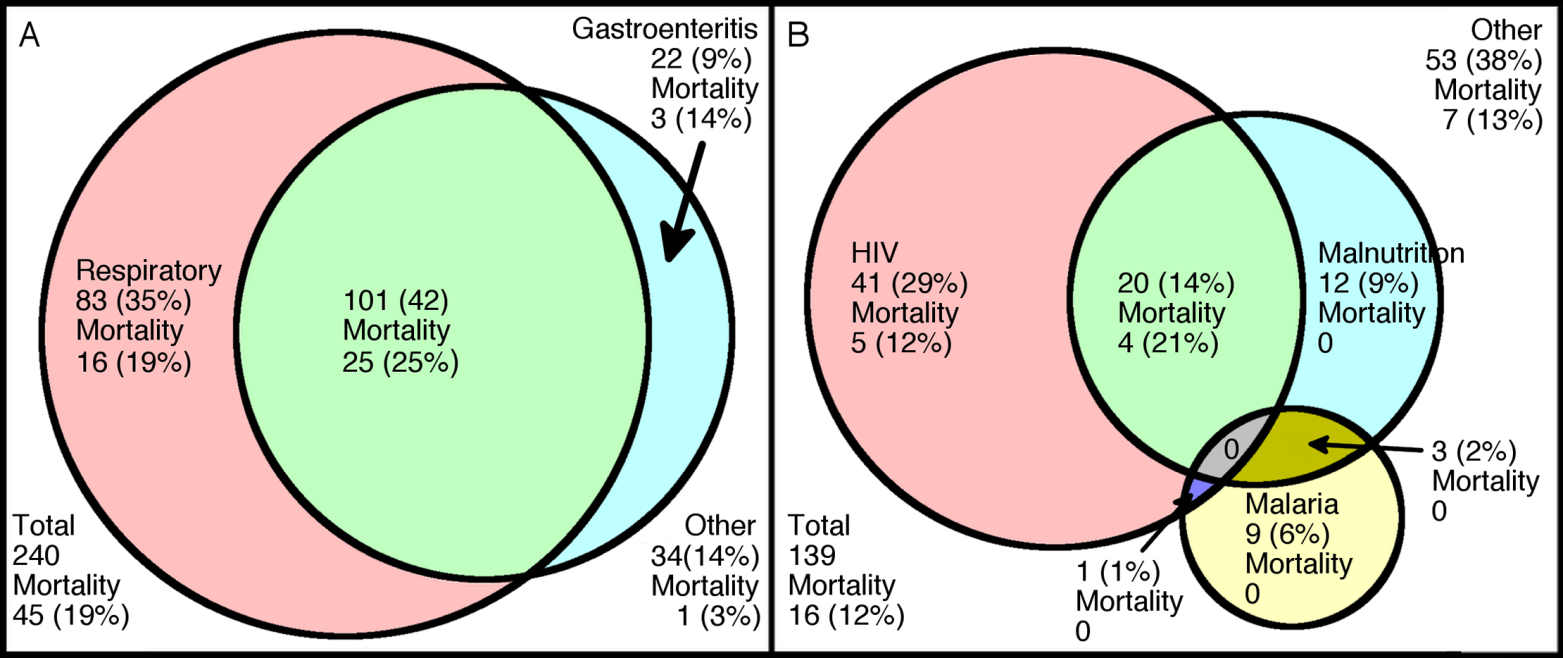
Clinical presentation and comorbidities of invasive *Salmonella* disease and mortality in Malawian children. (A) Area-proportionate Venn diagram indicating the different presenting clinical syndromes of pediatric invasive *Salmonella* disease. Gastroenteritis is defined as children with a history of diarrhea or vomiting, and a respiratory presentation as the presence of cough, shortness of breath or respiratory distress. (B) Area-proportionate Venn diagram indicating comorbidities (HIV, malaria and severe malnutrition) of children presenting with invasive *Salmonella* disease. Figures are absolute numbers (percentages in brackets) of children in each group, with mortality in each group given below and are shown for individual variables and for children in whom these overlapped. Discrepancies between totals in the figures and the text are due to children with missing data for one of the categories depicted.

Invasive *Salmonella* disease occurred mainly in children under three years of age and, as previously reported, was more common in the rainy season [8, 9, 21, 35]. In 206/240 (86%) of children, the disease presented with fever together with respiratory symptoms, gastrointestinal symptoms or both of these (Fig 4A). Since a clinical diagnosis of invasive *Salmonella* disease is not reliable, laboratory detection of *Salmonellae* from blood or CSF by microbiological culture is required. Facilities for this are uncommon in African hospitals and, even where present, a diagnosis usually takes a minimum of 48 hours. Despite NTS being primarily considered an enteric pathogen, the majority of children presented with cough or shortness of breath, commonly in the absence of enteric symptoms, as previously observed [8–10, 38].

This partly explains why the large majority of children with iNTS disease did not receive empirical antimicrobials with good anti-*Salmonella* activity on admission. Conversion to an antimicrobial regimen with adequate *Salmonella* cover occurred with a median delay of 3 days from presentation, by which time half of the observed deaths had occurred. Therefore, in settings where NTS are responsible for a large proportion of invasive pediatric bacterial infections, empirical antimicrobial therapy for suspected bacteremia and sepsis should take into account local antimicrobial sensitivity of NTS. There has been a decline in antimicrobial resistance among iNTS isolates in Malawi since this study took place, but the majority of *S*. Typhimurium were still multidrug resistance in 2014. A small number of *Salmonella* isolates with altered susceptibility to ciprofloxacin and 3^rd^-generation cephalosporins have been reported in recent years [39].

The rapid demise of Malawian children with iNTS disease is similar to findings from Kenya [9]. This is a major challenge for the management of children presenting with a syndrome indistinguishable from severe pneumonia, for which WHO guidelines recommend the use of ampicillin and chloramphenicol [38]. Early deaths also increase the difficulty of studying iNTS disease, as children will often die before a microbiological diagnosis of Gram-negative bacteremia or meningitis is possible. This potential confounder can only be overcome by recruiting every child at the time of admission. With up to 400 pediatric admissions a day at QECH, this was not a feasible option in our setting.

Even in clinical facilities with diagnostic laboratories and appropriate antimicrobial therapy, the burden of iNTS disease remains high. Until a vaccine against iNTS disease becomes available and widespread immunization of children across sub-Saharan Africa is implemented, development of a rapid diagnostic that can identify children with iNTS disease in a timely manner should remain a public health priority. First-generation tests would be unlikely to provide information on antimicrobial resistance. Multidrug resistance (defined as resistance to ampicillin, chloramphenicol and cotrimoxazole) is common among NTS blood culture isolates across Africa [40, 41]. Therefore, in countries where conventional microbiological surveillance and antimicrobial resistance testing are lacking, treatment of NTS in the absence of information on antimicrobial resistance should avoid these antibiotics.

The possible emergence of new antimicrobial resistance patterns underlies the ongoing need for blood culture surveillance data with antimicrobial resistance testing. Owing to the ability of *Salmonella* to sequester in the intracellular niche within macrophages and cause latent/recrudescent infection, antibiotics with good intracellular penetration, such as ciprofloxacin, are advisable. The lack of reported deaths in the four to six week follow-up period emphasizes the importance and value of providing intensive clinical care to children with iNTS disease through their acute admission.

Comorbidity was common, with 86/139 (62%) of children presenting with at least one of: *P*. *falciparum* parasitemia, severe malnutrition or HIV infection (Fig 4B). Only 38/249 (15%) had concurrent parasitemia, fewer than has been reported previously [8, 9]. In contrast to previous reports [21], this proportion did not vary significantly with season, suggesting other reasons for seasonal fluctuation in iNTS disease cases, such as worsening sanitation during the rainy season facilitating transmission. We did not test for recent malaria, e.g. by detection of HRP2 in plasma. Recent malaria can be more strongly associated with iNTS disease in children than current malaria [9]. Hemolysis-mediated immunological defects [42], which predispose children with malaria to iNTS disease, persist for several weeks following parasite clearance [43].

The dominant co-morbidity was HIV infection, affecting 70/162 (43%) of the children in the study. This greater proportion than has been reported previously [8, 9], likely reflects the systematic testing for HIV infection in the study. In other studies, HIV testing has often only been performed on a subset of children where HIV/AIDS has been suspected clinically (for example [1]). The significance of HIV infection as a risk factor for iNTS disease may vary across Africa, being more important in Southern and Eastern Africa compared with Western Africa owing to regional differences in HIV prevalence.

Background prevalence of HIV infection among children in Malawi at the time of the study was not known. However, available data of antenatal HIV prevalence and the numbers of children living with HIV in Malawi at the time of the study, suggest that the prevalence for the group under-five years was less than 2% [44]. Data on HIV status were only available for 161/263 (61%) of the children, so these findings should be viewed with some caution. Nevertheless, proportionately more children who died lacked HIV data (29/52, 56%), as compared to those who survived (66/204, 32%; p = 0.003 for comparison of proportions), and so the association of HIV with mortality may underestimate the prevalence of HIV co-infection.

This study aimed to identify the children in whom iNTS disease is most likely to be fatal. Children with HIV infection, those with a history of dyspnea, and children under 7 months of age are all at significantly increased risk of dying. The odds of dying with iNTS disease are 10 times greater in children under 7 months than they are in older children. This substantially-increased risk of mortality in young infants is particularly striking (Fig 3A) and is observed in spite of a significantly lower prevalence of HIV infection in this age group. Immune naivety is the most likely reason. Infants are at particular risk of iNTS disease after maternally-acquired antibody is lost and before they produce their own NTS-specific antibodies [45–47].

The dyspnea associated with increased risk of dying is most likely due to metabolic acidosis resulting from *Salmonella* sepsis, or to bona fide *Salmonella* respiratory tract infection. Arterial blood gas and anion gap measurements were not available, and chest radiographs not commonly performed in these children. Hence, it was not possible to distinguish the underlying pathological mechanism. A history of shortness of breath often results in a child being misclassified as suffering from pneumonia in resource-poor healthcare settings [38], increasing the chance of children in this high risk subgroup receiving inappropriate empirical antimicrobials.

Although the association between HIV and iNTS disease is well established, the underlying mechanisms of HIV infection as a determinant of mortality in children with iNTS disease has not been well explored. Immunological defects in HIV-infected adults leading to susceptibility to iNTS disease include impaired gut mucosal immunity [48], and dysregulated cellular [49] and humoral immunity [50, 51]. The mechanisms that contribute to increased mortality in children with iNTS disease and HIV provide important questions for future research, which should explore the key interaction of HIV infection and malnutrition [31].

Only three children at presentation were receiving anti-retroviral therapy (ART), while 37 were on CPT. CPT has been shown to protect against iNTS disease in HIV-infected African adults prior to the use of ART [52] and subsequent studies have shown benefit of CPT in protecting against invasive bacterial disease in communities with high levels of in vitro cotrimoxazole resistance [53]. However, CPT did not affect outcome in our study among HIV-infected children with iNTS disease. Recent work has indicated an important role for the introduction of ART among HIV-infected African children in reducing the incidence of iNTS disease [26, 31], but has yet to show that ART improves outcome among HIV-infected children with iNTS disease [31].

### Limitations

The main limitation of the study is that these data were collected from one site during one period in time, the calendar year 2006. New prospective studies from other sites in sub-Saharan Africa would be valuable for confirming the reproducibility of our findings across the region and their ongoing applicability to iNTS disease in Africa. In Blantyre, the epidemiology of invasive *Salmonella* disease changed markedly in 2011 with the start of an epidemic of typhoid fever. By 2012 *S*. Typhi was a more common blood culture isolate than NTS [39], indicating the fluidity of the epidemiology of invasive *Salmonella* disease in one site.

Additionally, iNTS disease has declined in Blantyre, accompanied by reductions in malaria, HIV (following the successful implementation of an antiretroviral therapy program beginning in 2005) and acute malnutrition [31]. Nevertheless, across sub-Saharan Africa, iNTS disease remains the commonest presentation of invasive *Salmonella* disease and a major cause of pediatric bacteremia [2,8,27].

## Conclusions

This prospective study provides additional evidence that iNTS disease is common and frequently fatal among children in sub-Saharan Africa, and particularly highlights the major diagnostic and management challenges associated with iNTS disease [7]. iNTS disease does not present with a readily-identifiable clinical syndrome and current empirical antimicrobials do not provide effective treatment for *Salmonella* infections. Empirical antimicrobial treatment for potential *Salmonella* infection should be considered for children presenting with a clinical syndrome compatible with systemic infection. This is especially true for children with HIV infection and young infants, and particularly applies to presentations suggestive of community-acquired pneumonia in regions where invasive *Salmonella* disease is common.

The development of cheap rapid diagnostic tests for iNTS disease in children should be a priority. The poor effectiveness of antimicrobials means that other approaches to treatment are required. Epidemiological studies provide strong evidence that antibodies bactericidal to iNTS in the presence of complement protect against these infections [45, 46]. The development and use of a vaccine that induces protective antibodies in young infants may provide effective protection [6, 7].

## Supporting information

S1 Checklist STROBE checklist. (DOC)

S1 FigDistribution and influence of residuals in logistic regression model of mortality.The graph demonstrates for each covariate pattern the squared standardized Pearson residual (on the y axis) and the probability of mortality for an individual with that covariate pattern (x axis). The area of each data point is proportional to $\Delta {\hat{\beta }}_{j}$ influence statistic. The graph demonstrates no relationship between the probability of mortality for a given covariate pattern and the magnitude of the residuals. Residual analysis of the 14 covariate patterns demonstrates a single covariate pattern with a squared Pearson standardized residual greater than 3.84 (95% significance level–horizontal line on graph) with a $\Delta {\hat{\beta }}_{j}$ influence statistic of 0.44 indicating the model is consistent with the magnitude and influence of the residuals.(DOCX)

S1 Table Clinical data spreadsheet. (XLS)
