# Supplementary material for: Presentation of life-threatening invasive nontyphoidal Salmonella disease in Malawian children: A prospective observational study
Source: PLoS Negl Trop Dis. 2017 Dec 7;11(12):e0006027. doi: 10.1371/journal.pntd.0006027 (PMC5745124; doi:10.1371/journal.pntd.0006027)
Supplement: S1 Checklist — (DOC) [file pntd.0006027.s001.doc]

STROBE Statement—Checklist of items that should be included in reports of ***cross-sectional studies***

|  | Item No | Recommendation |
| --- | --- | --- |
| **Title and abstract** | 1 | (*a*) Indicate the study’s design with a commonly used term in the title or the abstract  ‘Prospective Cross-sectional Observational Study’, p1 |
|  |  | (*b*) Provide in the abstract an informative and balanced summary of what was done and what was found  See Abstract, p4 |
| Introduction | | |
| Background/rationale | 2 | Explain the scientific background and rationale for the investigation being reported  See Background, p6-7. Nontyphoidal Salmonella is a common cause of bacteremia in Africa, but there is a lack of prospective studies of the disease that can be used to guide improved management |
| Objectives | 3 | State specific objectives, including any pre-specified hypotheses  See Background, p6-7. Consecutive microbiologically-confirmed cases admitted to a large government hospital in Malawi were studied for the relationship between clinical presentation and outcome. |
| Methods | | |
| Study design | 4 | Present key elements of study design early in the paper  See Methods: Study patients and clinical methods, p8 |
| Setting | 5 | Describe the setting, locations, and relevant dates, including periods of recruitment, exposure, follow-up, and data collection  See Methods: Study site, Study patients and clinical methods, p8. Specified location – Queen Elizabeth Central Hospital, Blantyre, Malawi. Specified period of recruitment – Jan 1 to Dec 31 2006 |
| Participants | 6 | (*a*) Give the eligibility criteria, and the sources and methods of selection of participants  See Methods: Study patients and clinical methods, p8. Eligibility criteria were age ≤ 14 years, admission to QECH and isolation of *Salmonella* from blood and/or CSF in the calendar year of 2006. Subjects not meeting all three eligibility criteria were excluded. |
| Variables | 7 | Clearly define all outcomes, exposures, predictors, potential confounders, and effect modifiers. Give diagnostic criteria, if applicable  See Methods: Study patients and clinical methods, p8-9. Outcomes – Clinical progress was recorded daily through to discharge from hospital or inpatient death. Exposures, predictors, potential confounders and effect modifiers were defined in relation to clinical presentation (e.g. respiratory distress, gastroenteritis, fever), comorbidities (e.g. malnutrition, HIV infection, malaria), age (since iNTS disease is most prevalent among children under two years and season (since incidence of iNTS disease is highest during the rains). Diagnostic criteria: isolation of *Salmonella* from blood and/or CSF. |
| Data sources/ measurement | 8* | For each variable of interest, give sources of data and details of methods of assessment (measurement). Describe comparability of assessment methods if there is more than one group  See Methods: Study patients and clinical methods, and Sampling and laboratory materials, p8-9. |
| Bias | 9 | Describe any efforts to address potential sources of bias  See Methods: Study patients and clinical methods, p8. In order to reduce bias due to early death following admission, children were approached for recruitment on the day that Gram-negative bacteria were first detected in the blood or CSF. |
| Study size | 10 | Explain how the study size was arrived at  See Methods: Study patients and clinical methods, p8. Study size was simply a function of the number of consecutive children admitted to QECH in 2006 with *Salmonella* bacteremia and/or meningitis. |
| Quantitative variables | 11 | Explain how quantitative variables were handled in the analyses. If applicable, describe which groupings were chosen and why  See Methods: Statistical analysis, p10. Data were entered into a Microsoft Access database and analysis was performed using R. Further details follow in text. |
| Statistical methods | 12 | (*a*) Describe all statistical methods, including those used to control for confounding  See Methods: Statistical analysis, p10. Variation with age and season, of clinical indices, co-morbidity and outcome, were calculated as Mantel-Haenszel odds ratios. Odds ratios of case fatality given the presence of clinical indices or co-morbidity adjusted for age and season of presentation were calculated by logistic regression. Finally, a model of case fatality was constructed and tested by multivariate logistic regression. Further details follow in text. |
|  |  | (*b*) Describe any methods used to examine subgroups and interactions  See Methods: Statistical analysis, p10. As above |
|  |  | (*c*) Explain how missing data were addressed  See Methods: Statistical analysis, p10. Missing data were not considered in the analysis and were addressed by providing denominator information, where applicable. |
|  |  | (*d*) If applicable, describe analytical methods taking account of sampling strategy  Not applicable |
|  |  | (*e*) Describe any sensitivity analyses  Not applicable |
| Results | | |
| Participants | 13* | (a) Report numbers of individuals at each stage of study—eg numbers potentially eligible, examined for eligibility, confirmed eligible, included in the study, completing follow-up, and analysed  See Results, p11 and flow chart, Figure 1 |
|  |  | (b) Give reasons for non-participation at each stage  See Results, p11 and flow chart in Figure 1 |
|  |  | (c) Consider use of a flow diagram  See flow chart, Figure 1 |
| Descriptive data | 14* | (a) Give characteristics of study participants (eg demographic, clinical, social) and information on exposures and potential confounders  See Results, p11, Tables 1 Characteristics of children presenting with invasive *Salmonella* disease, Table 2, Clinical characteristics of Malawian children with invasive *Salmonella* disease and associated mortality and Figure 2 Variability in outcome, co-morbidity and clinical presentation of invasive *Salmonella* disease in Malawian children with age and season of presentation |
|  |  | (b) Indicate number of participants with missing data for each variable of interest  See Results, p11, Tables 1 Characteristics of children presenting with invasive *Salmonella* disease, Table 2, Clinical characteristics of Malawian children with invasive *Salmonella* disease and associated mortality. Numbers of participants with missing data for each variable of interest are indicated by provision of denominators for each variable. |
| Outcome data | 15* | Report numbers of outcome events or summary measures  See Results, Mortality, p14, Tables 1 Characteristics of children presenting with invasive *Salmonella* disease (Inpatient mortality), and Table 2, Clinical characteristics of Malawian children with invasive *Salmonella* disease and associated mortality. |
| Main results | 16 | (*a*) Give unadjusted estimates and, if applicable, confounder-adjusted estimates and their precision (eg, 95% confidence interval). Make clear which confounders were adjusted for and why they were included  See Results, p11-13. 95% confidence intervals provided with each risk estimate. Age and season were adjusted for since inpatient mortality varied significantly with age, and frequency of disease varies markedly with season in Malawi (most common in the rains). |
|  |  | (*b*) Report category boundaries when continuous variables were categorized  See Methods, Statistical Analysis, p10. Variation with age and season, of clinical indices, co-morbidity and outcome, were calculated as Mantel-Haenszel odds ratios. To facilitate this, age was converted into four ordinal categories (0-6 months, 7-12 months, 1-2 years, >2 years). Presentation season was grouped as January-March, April-June, July-September and October-December and ranked by mean rainfall. |
|  |  | (*c*) If relevant, consider translating estimates of relative risk into absolute risk for a meaningful time period  Not applicable |
| Other analyses | 17 | Report other analyses done—eg analyses of subgroups and interactions, and sensitivity analyses  See Results, p16. A multivariate model predicting mortality including age, sex, HIV status and a history of dyspnea was shown to be statistically significant (likelihood ratio test χ^2^=21.69, p= 0.0002) with a pseudo-R^2^ of 0.179. The Hosmer-Lemeshow goodness-of-fit test for the model provides no evidence to reject the model (p=0.73) and the distribution and influence of the residuals (Figure S1) demonstrate that the model is consistent with the data. |
| Discussion | | |
| Key results | 18 | Summarise key results with reference to study objectives  See Discussion, p20. This study aimed to identify the children in whom iNTS disease is most likely to be fatal. Children with HIV infection, those with a history of dyspnea, and children under 7 months of age are all at significantly increased risk of dying. |
| Limitations | 19 | Discuss limitations of the study, taking into account sources of potential bias or imprecision. Discuss both direction and magnitude of any potential bias  See Discussion, p17-20. Early deaths also increase the difficulty of studying iNTS disease, as children will often die before a microbiological diagnosis of Gram-negative bacteremia or meningitis is possible. This potential confounder can only be overcome by recruiting every child at the time of admission. Data on HIV status were only available for 161/263 (61%) of the children, so these findings should be viewed with some caution. Nevertheless, proportionately more children who die lack HIV data (29/52, 56%), as compared to those who survive (66/204, 32%; p=0.003 for comparison of proportions), and so the association of HIV with mortality may underestimate the prevalence of HIV co-infection. |
| Interpretation | 20 | Give a cautious overall interpretation of results considering objectives, limitations, multiplicity of analyses, results from similar studies, and other relevant evidence  See Abstract, p4. Young infants, children with dyspnea and HIV-infected children bear a disproportionate burden of the *Salmonella*-associated mortality in Malawi. Strategies to improve prevention, diagnosis and management of invasive *Salmonella* disease should be targeted at these children. |
| Generalisability | 21 | Discuss the generalisability (external validity) of the study results  See Discussion, p17-21. Generalisability across Africa, particularly with reference to the dominance of HIV as a comorbidity and risk factor for increased mortality, will depend on prevalence of key factors such as HIV infection: The significance of HIV infection as a risk factor for iNTS disease may vary across Africa, being more important in Southern and Eastern Africa compared with Western Africa owing to regional differences in HIV prevalence. |
| Other information | | |
| Funding | 22 | Give the source of funding and the role of the funders for the present study and, if applicable, for the original study on which the present article is based  Funding information including role of the funders is included in the online system for submissions to PLoS Negl Trop Dis. |

*Give information separately for exposed and unexposed groups.

**Note:** An Explanation and Elaboration article discusses each checklist item and gives methodological background and published examples of transparent reporting. The STROBE checklist is best used in conjunction with this article (freely available on the Web sites of PLoS Medicine at http://www.plosmedicine.org/, Annals of Internal Medicine at http://www.annals.org/, and Epidemiology at http://www.epidem.com/). Information on the STROBE Initiative is available at www.strobe-statement.org.
