# Supplementary material for: Presentation of life-threatening invasive nontyphoidal Salmonella disease in Malawian children: A prospective observational study
Source: PLoS Negl Trop Dis. 2017 Dec 7;11(12):e0006027. doi: 10.1371/journal.pntd.0006027 (PMC5745124; doi:10.1371/journal.pntd.0006027)
Supplement: S1 Fig — The graph demonstrates for each covariate pattern the squared standardized Pearson residual (on the y axis) and the probability of mortality for an individual with that covariate pattern (x axis). The area of each data point is proportional to Δβ^j influence statistic. The graph demonstrates no relationship between the probability of mortality for a given covariate pattern and the magnitude of the residuals. Residual analysis of the 14 covariate patterns demonstrates a single covariate pattern with a squared Pearson standardized residual greater than 3.84 (95% significance level–horizontal line on graph) with a Δβ^j influence statistic of 0.44 indicating the model is consistent with the magnitude and influence of the residuals. (DOCX) [file pntd.0006027.s002.docx]

**Figure S1. Distribution and influence of residuals in logistic regression model of mortality.**

The graph demonstrates for each covariate pattern the squared standardized Pearson residual (on the y axis) and the probability of mortality for an individual with that covariate pattern (x axis). The area of each data point is proportional to $\Delta\hat{\beta}$_j_ influence statistic. The graph demonstrates no relationship between the probability of mortality for a given covariate pattern and the magnitude of the residuals. Residual analysis of the 14 covariate patterns demonstrates a single covariate pattern with a squared Pearson standardized residual greater than 3.84 (95% significance level – horizontal line on graph) with a $\Delta\hat{\beta}$_j_ influence statistic of 0.44 indicating the model is consistent with the magnitude and influence of the residuals.
